# Supplementary material for: Accelerated lysine metabolism conveys kidney protection in salt-sensitive hypertension
Source: Nat Commun. 2022 Jul 14;13:4099. doi: 10.1038/s41467-022-31670-0 (PMC9283537; doi:10.1038/s41467-022-31670-0)
Supplement: Supplementary file 1 — Supplementary Information [file 41467_2022_31670_MOESM1_ESM.pdf]

Supplementary methods file.

1. **Synthesis of analytical standards**

General

All reagents and solvents were purchased from commercial suppliers and used without further purification. NMR spectra were recorded on a Bruker AVIII (600 MHz) spectrometer at ambient temperature. Chemical shifts (d) of ^{1}H NMR spectra are reported in ppm with the solvent resonance employed as internal standard ($CHCl_3$ at 7.26 ppm). Peaks are reported as s = singlet, d = doublet, t = triplet, q = quartet, m = multiplet or unresolved, br = broad signal, coupling constant(s) in Hz (*J*), integration. ^{13}C NMR spectra were recorded with ^{1}H-decoupling and chemical shifts are reported in ppm with the solvent resonance employed as internal standard ($CDCl_3$ at 77.16 ppm). Purification by mass directed preparative reversed phase HPLC was performed on a Waters Autopurification LC with a Waters BEH C18 column (5 um, 19x160 mm) using a 0.1% aqueous formic acid:acetonitrile gradient (30 mL/min, main segment of gradient at 5-20% acetonitrile over 8 minutes) at ambient temperature. Fractionation was triggered by a Waters QDa single quadrupole mass spec (ESI+).

Synthesis of *N*^{6}-malonyl-*L*-lysine 1

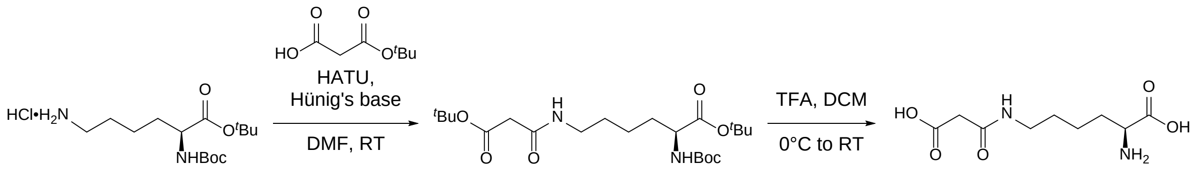

Boc-Lys-OtBu•HCl **1a** **1** *N*^{6}-malonyl-*L*-lysine

To a solution of Boc-Lys-OtBu·HCl (51 mg, 0.15 mmol, 1.0 equiv) and malonic acid mono-tert-butyl ester (29 mg, 0.18 mmol, 1.2 equiv) in DMF (0.75 mL) was added HATU (39 mg, 0.17 mmol, 1.1 equiv), followed by triethylamine (65 mL, 0.38 mmol, 2.5 equiv). The reaction was stirred at room temperature for 30 min, subsequently diluted with brine (15 mL) and extracted with EtOAc. The combined organic phase was washed with 5% aqueous LiCl solution, dried over Na_2SO_4 and concentrated under reduced pressure. The crude product was purified by flash column chromatography (hexanes/EtOAc 5:1 to 2:1) to afford **1a** (55 mg, 82%) as a colorless oil.

Analytical standard **1** for comparison with metabolite extracts from tissue was prepared by deprotection of **1a** (22.3 mg, 0.05 mmol, 1.0 equiv) using TFA/DCM (1:1, 1 mL) for 2 hours at room temperature. Volatiles were removed under reduced pressure and the crude product was purified by mass directed preparative reversed phase HPLC to give the formic acid salt of **1** (11 mg, 79%) after lyophilization which was directly used as an analytical standard after resuspension in acetonitril:water 1:1 and characterization by MS/MS.

Analytical data of 1a: **^{1}H NMR** (600 MHz, $CDCl_3$) δ = 7.24 (br, 1H), 5.06 (d, *J* = 8.4 Hz, 1H), 4.17 (q, *J* = 7.4, 7.0 Hz, 1H), 3.29 (td, *J* = 7.1, 5.6 Hz, 2H), 3.22 (s, 2H), 1.86 – 1.75 (m, 1H), 1.67 – 1.61 (m, 1H), 1.61 – 1.54 (m, 2H), 1.49 (s, 9H), 1.48 (s, 9H), 1.46 (s, 9H), 1.44 – 1.32 (m, 2H); **^{13}C NMR** (151 MHz, $CDCl_3$) δ = 172.0, 169.2, 165.6, 155.6, 82.6, 82.0, 79.8, 53.9, 42.1, 39.4, 32.8, 29.2, 28.5, 28.2, 28.1, 22.7; **HR-MS-ESI-TOF (*m/z*):** calculated $[M+H]^+$ for $C_{22}H_{41}N_2O_7$: 445.2908, measured 445.2908

Synthesis of N^2-malonyl-*L*-lysine

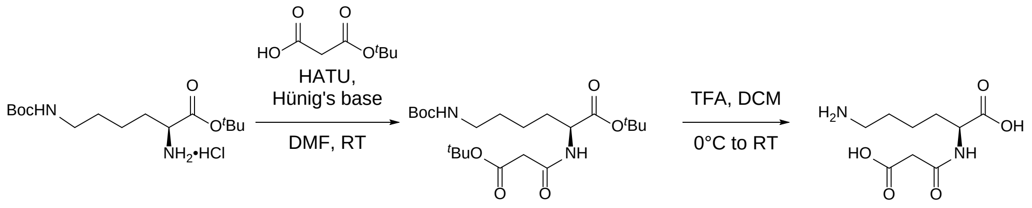

H-Lys(Boc)-OtBu•HCl **2a** **2** N^2-malonyl-*L*-lysine

To a solution H-Lys(Boc)-OtBu·HCl (51 mg, 0.15 mmol, 1.0 equiv) and malonic acid mono-tert-butyl ester (29 mg, 0.18 mmol, 1.2 equiv) in DMF (0.75 mL) was added HATU (39 mg, 0.17 mmol, 1.1 equiv), followed by triethylamine (65 μL, 0.38 mmol, 2.5 equiv). The reaction was stirred at room temperature for 30 min, subsequently diluted with brine (15 mL) and extracted with EtOAc. The combined organic phase was washed with 5% aqueous LiCl solution, dried over Na_2SO_4 and concentrated under reduced pressure. The crude product was purified by flash column chromatography (hexanes/EtOAc 5:1 to 2:1) to afford **2a** (51 mg, 76%) as a colorless oil.

Analytical standard **2** for comparison with metabolite extracts from tissue (extracted in was prepared by deprotection of **2a** (22.3 mg, 0.05 mmol, 1.0 equiv) using TFA/DCM (1:1, 1 mL) for 2 hours at room temperature. Volatiles were removed under reduced pressure and the crude product was purified by mass directed preparative reversed phase HPLC to give the formic acid salt of **2** (10 mg, 72%) after lyophilization which was directly used as an analytical standard after resuspension in acetonitril:water 1:1 and characterization by MS/MS.

Analytical data of 2a: **^{1}H NMR** (600 MHz, $CDCl_3$) δ = 7.56 (d, *J* = 7.6 Hz, 1H), 4.56 (br, 1H), 4.50 (td, *J* = 7.4, 5.2 Hz, 1H), 3.24 (s, 2H), 3.10 (d, *J* = 6.7 Hz, 2H), 1.88 – 1.81 (m, 1H), 1.73 – 1.65 (m, 1H), 1.54 – 1.49 (m, 2H), 1.48 (s, 9H), 1.47 (s, 9H), 1.43 (s, 9H), 1.41 – 1.28 (m, 2H); **^{13}C NMR** (151 MHz, $CDCl_3$) δ = 171.3, 168.5, 165.3, 156.1, 82.7, 82.3, 79.2, 52.7, 42.5, 40.4, 32.4, 29.7, 28.6, 28.2, 28.1, 22.4; **HR-MS-ESI-TOF (*m/z*):** calculated $[M+H]^+$ for $C_{22}H_{41}N_2O_7$: 445.2908, measured 445.2915

N^{6}-malonyl-*L*-lysine 1a

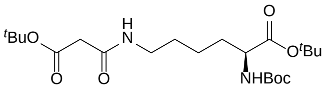

1a

^{1}H NMR: 600 MHz, $CDCl_3$

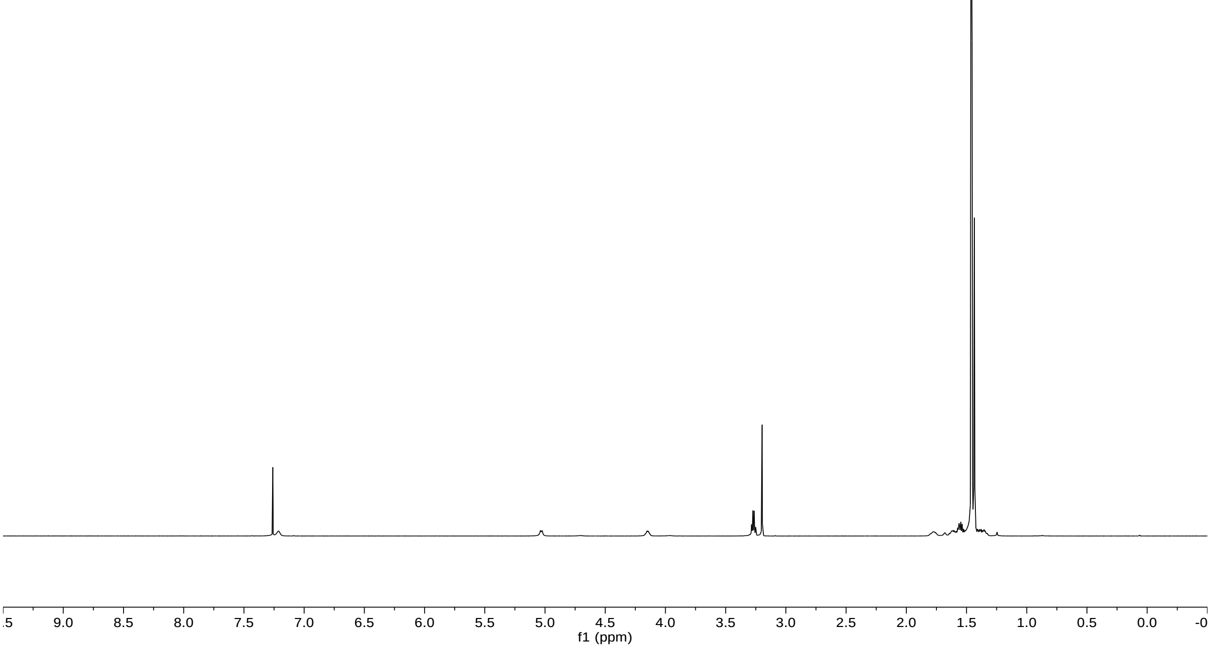

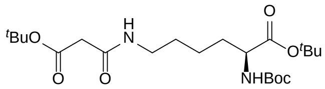

1a

^{13}C NMR: 151 MHz, $CDCl_3$

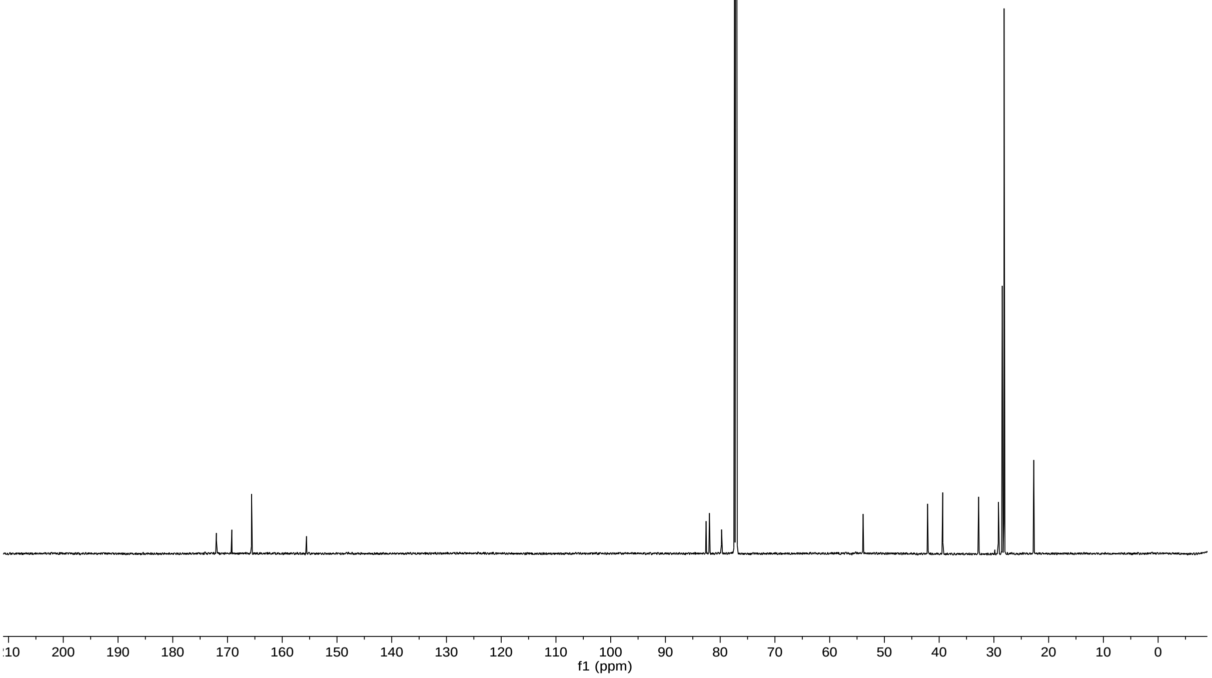

N^2-malonyl-*L*-lysine 2a

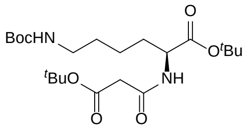

2a

^{1}H NMR: 600 MHz, $CDCl_3$

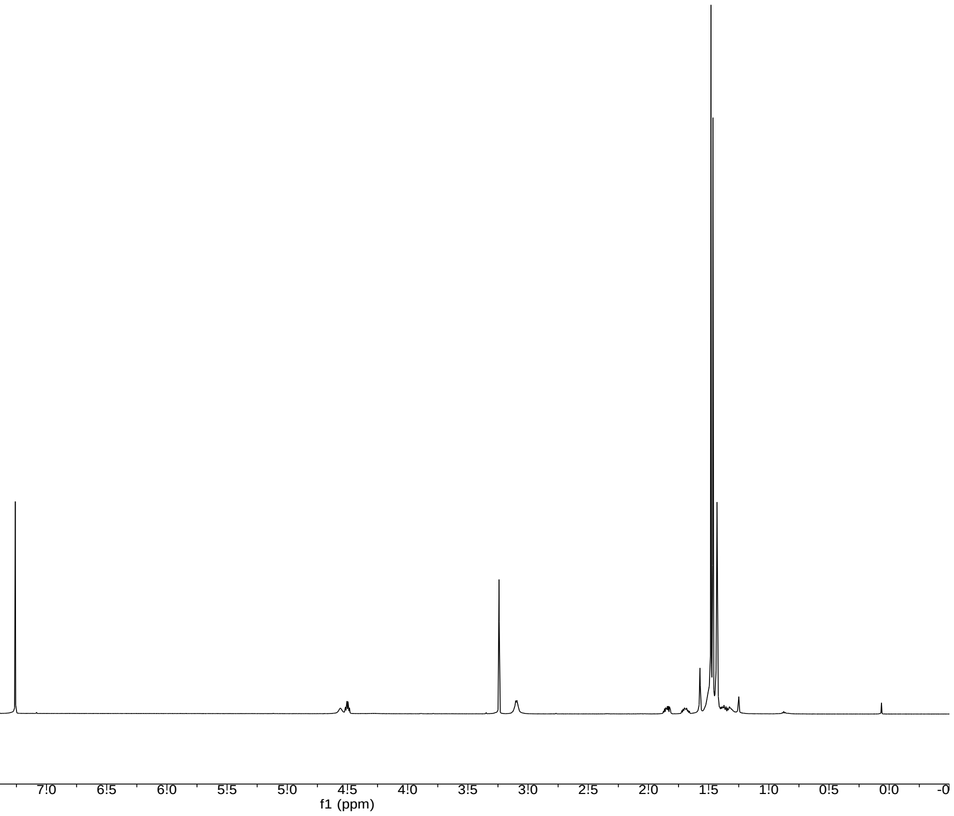

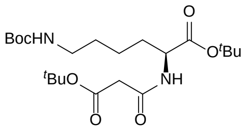

2a

^{13}C NMR: 151 MHz, $CDCl_3$

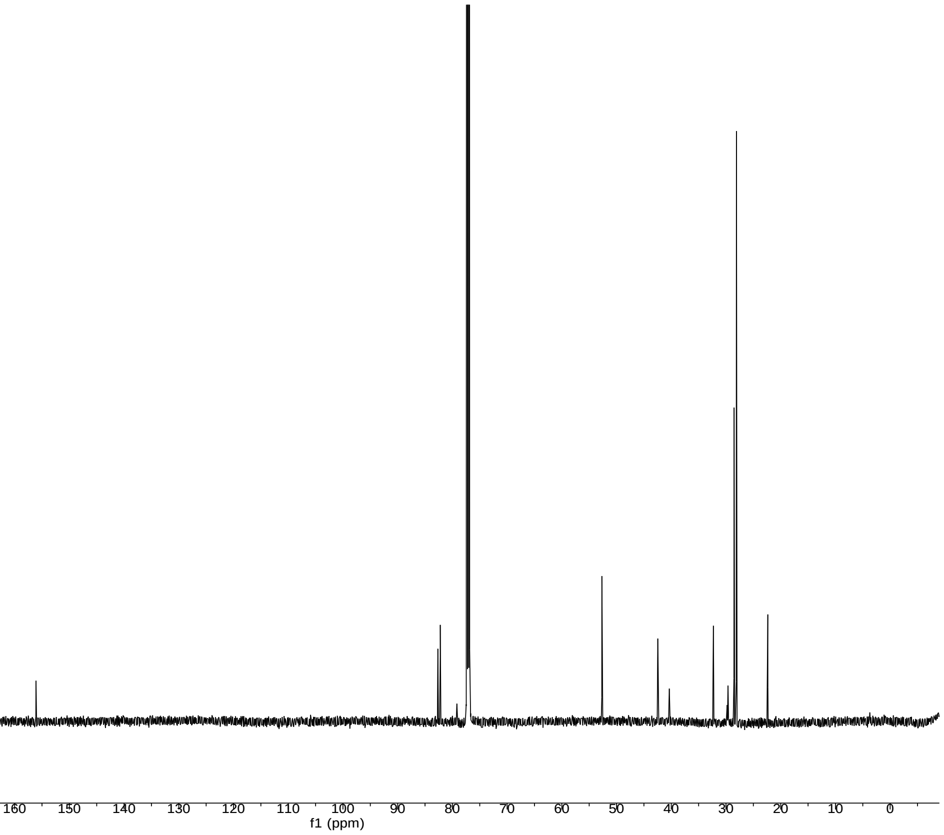

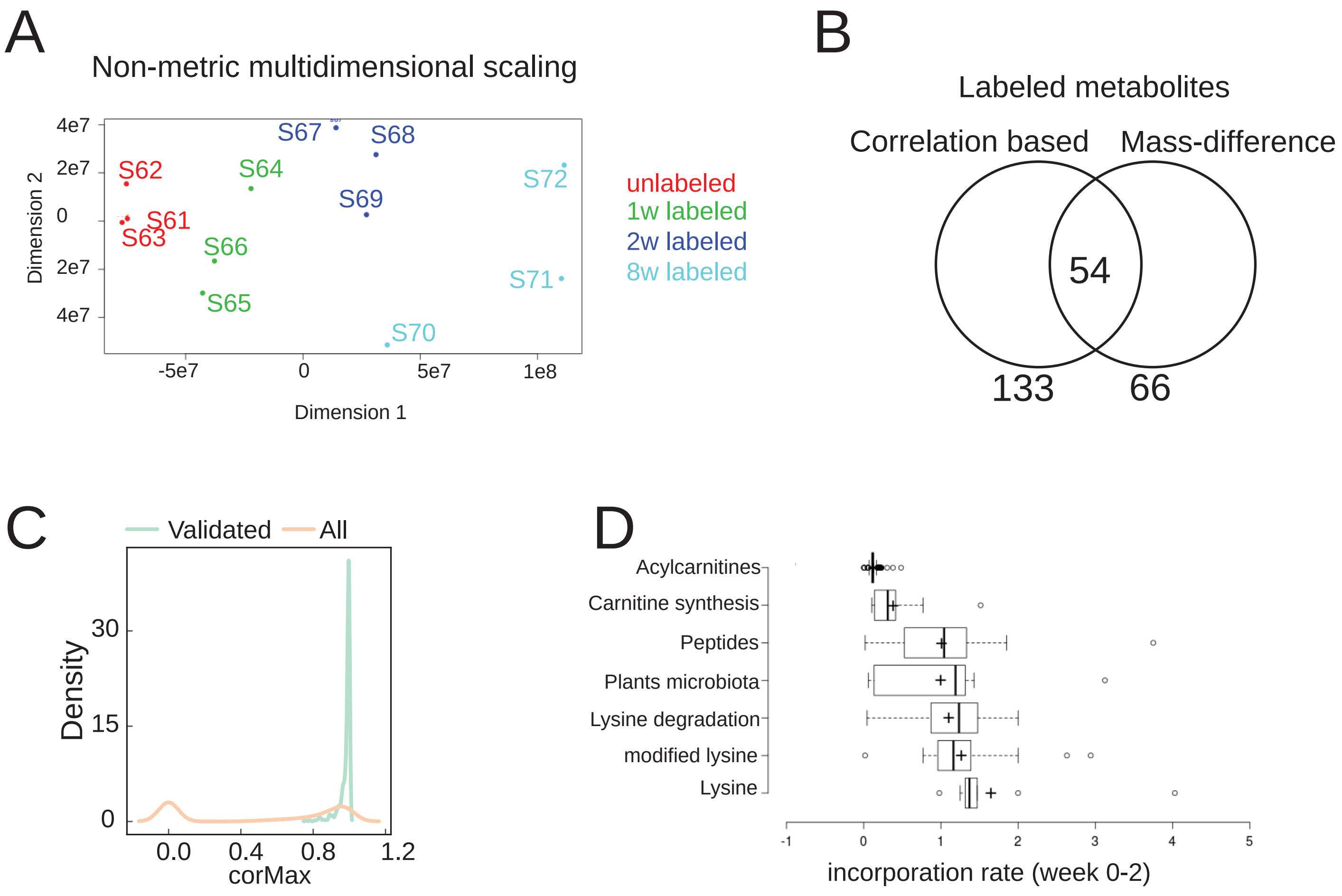

E

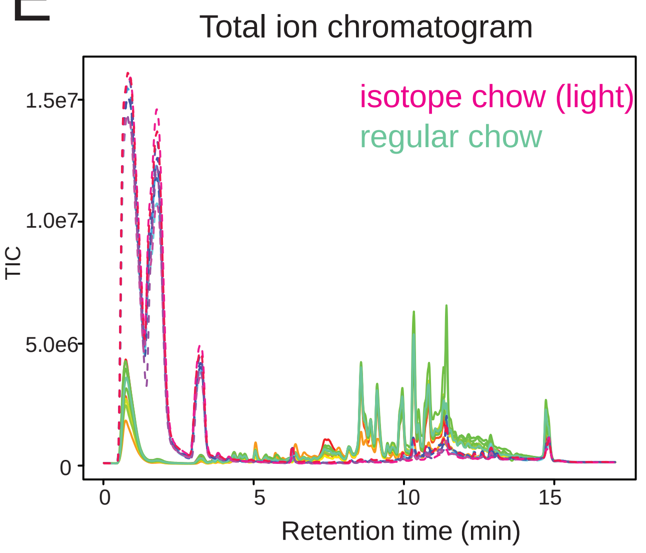

Metabolite	mass	normal chow	isotope chow
Lysine	147.1127	1873691	13316916.73
Carboxypropyltrimethylammonium	146.1173	n	n
Trimethyllysine	189.1595	n	n
Carnitine	162.11	n	n
Acetylcarnitine	204.1222	n	n
Propionylcarnitine	218.1382	n	n
Butyryl carnitine	232.1542	n	n
Hexanoylcarnitine	260.1856	n	n
Octanoylcarnitine	288.198	n	n
Decanoyl-carnitine	316.2306	n	n
Dodecanoylcarnitine, confirmed	344.2797	n	n
Myristoylcarnitine	372.3112	n	n
Palmitoylcarnitine	400.3406	n	n
Stearoylcarnitine	428.3732	n	n
Linoleoylcarnitine	424.3426	n	n
Elaidic carnitine	426.3576	n	n
arachidonoylcarnitine	448.3427	n	n
Arachidyl carnitine	456.4053	n	n

Supplemental Fig. 1: Supportive evidence for $^{13}C_6$ lysine metabolomic trace. A. Example for Non-metric multidimensional scaling of an isotope labeled sample reveals clear separation of 0, 1, 2 and 8 weeks labeled samples. **B.** Comparison of detected metabolites in mass-difference and correlation based approach. **C.** Density of all peaks and validated peaks in correlation based approach. **D.** Slope of incorporation across metabolite classes. **E.** Isotope labeled food metabolome as compared to normal chow. Carnitines and other lysine metabolites were not detected in the modified isotope labeled diet.

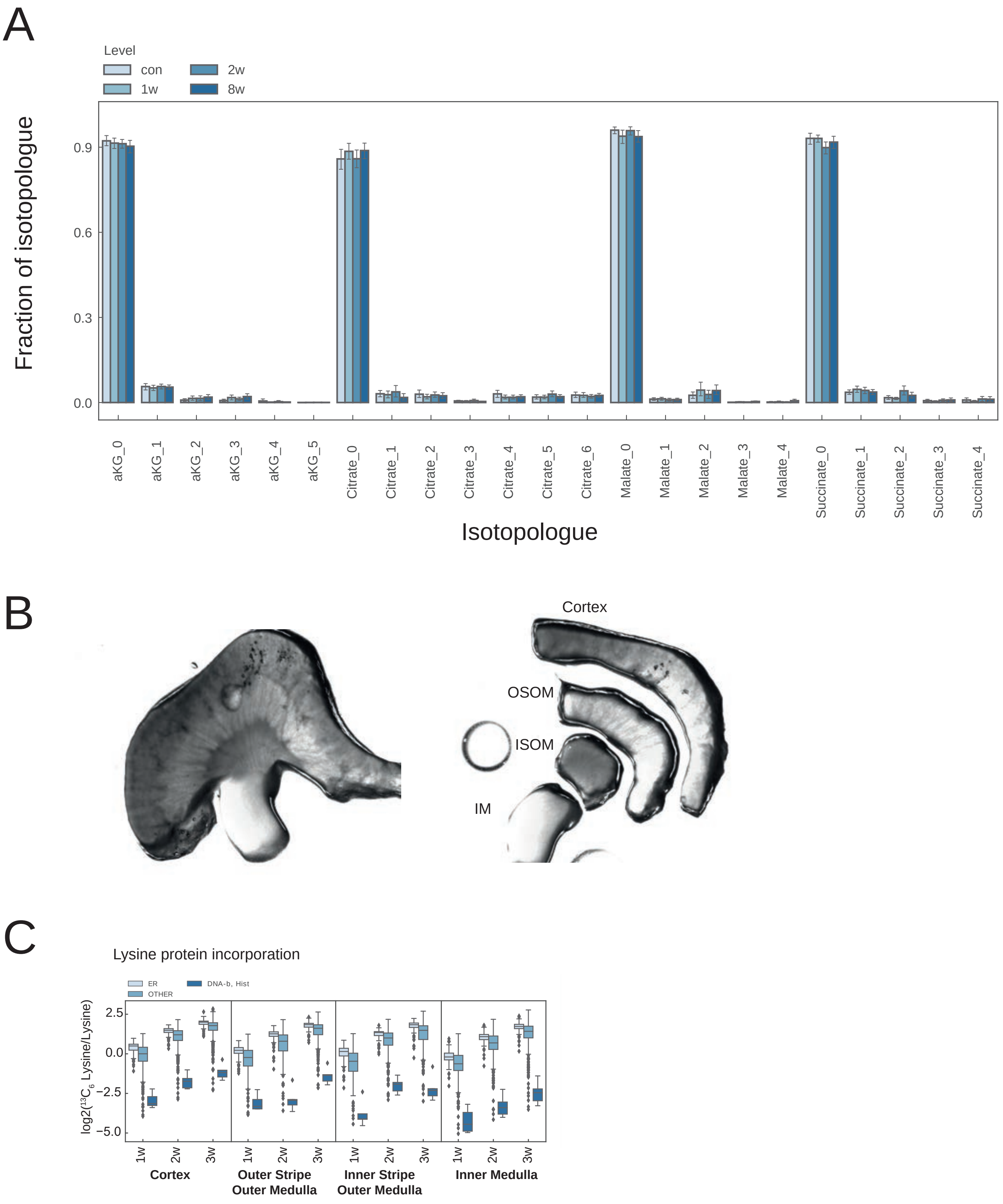

Supplemental Fig. 2: Analysis of lysine isotope incorporation in metabolites and proteins. A. Isotopologues of the TCA cycle were measured by targeted metabolomics in extracts from kidney cortex. No significant differences in incorporation and isotopologue pattern was observed (n= 3 independent animals, two-tailed t-test), error bars = SD. **B**. Dissection of kidneys. **C**. $^{13}C_6$ lysine is incorporated more rapidly into the proteins of the cortex, as compared to the inner stripe outer medulla or medulla. $^{13}C_6$ H/L ratio in proteins in different areas of the kidney.

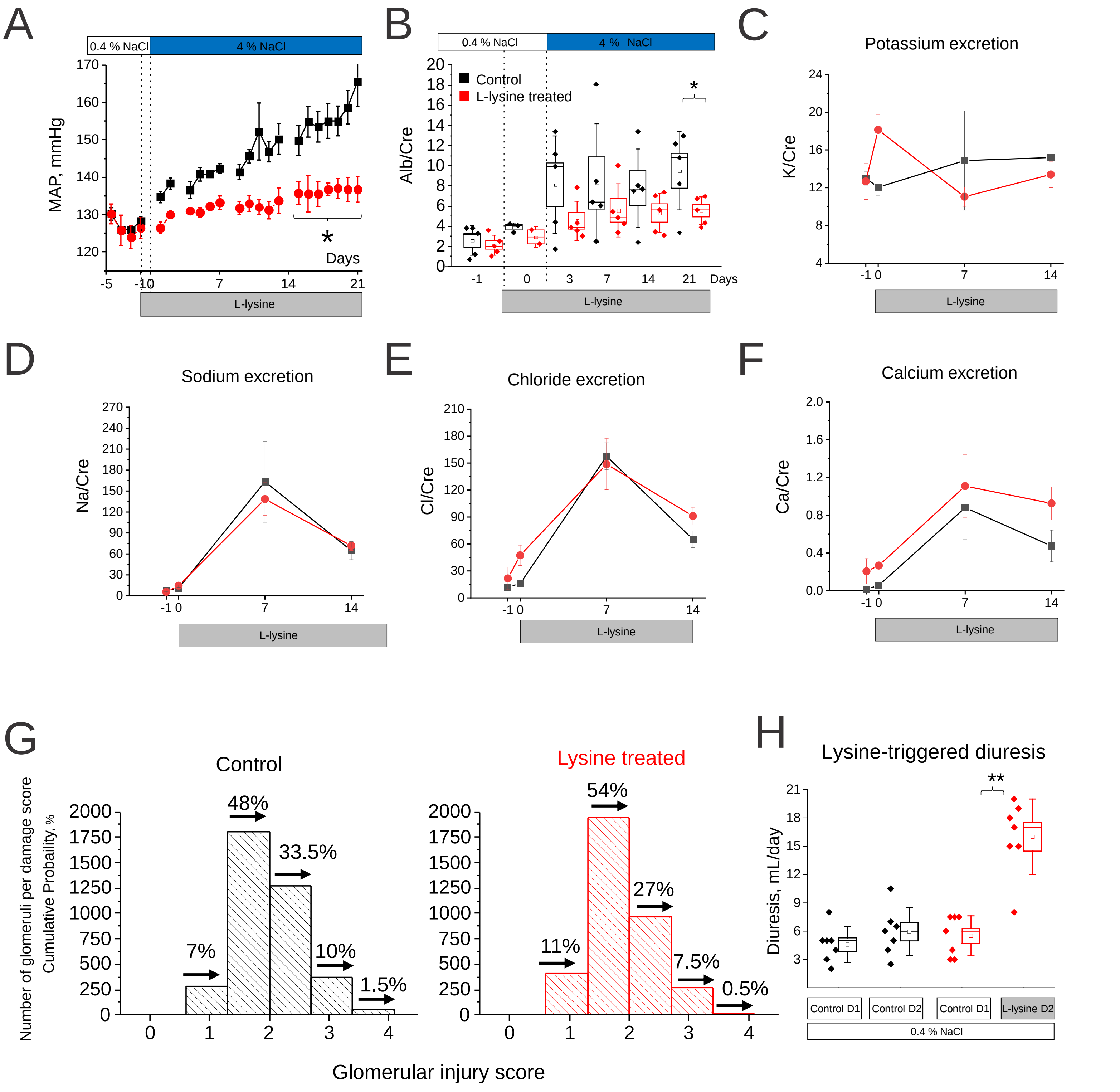

Supplemental Fig. 3: Extended phenotyping of lysine-treated Dahl SS rats. A. Mean arterial pressure (MAP) in female D/SS rats under L-lysine treatment. N= 5 independent animals, error bars = SEM **B.** Lysine treatment and proteinuria in the same groups as in **A**. **C-F.** Urinary potassium, sodium, chloride, and calcium excretion as a measure of sodium uptake. **G.** Neuronal net analysis of glomerular injury score in control and lysine treated SS rats. **H.** Effect of L-lysine on diuresis in SS rats when animals were fed normal (0.4% NaCl) diet.

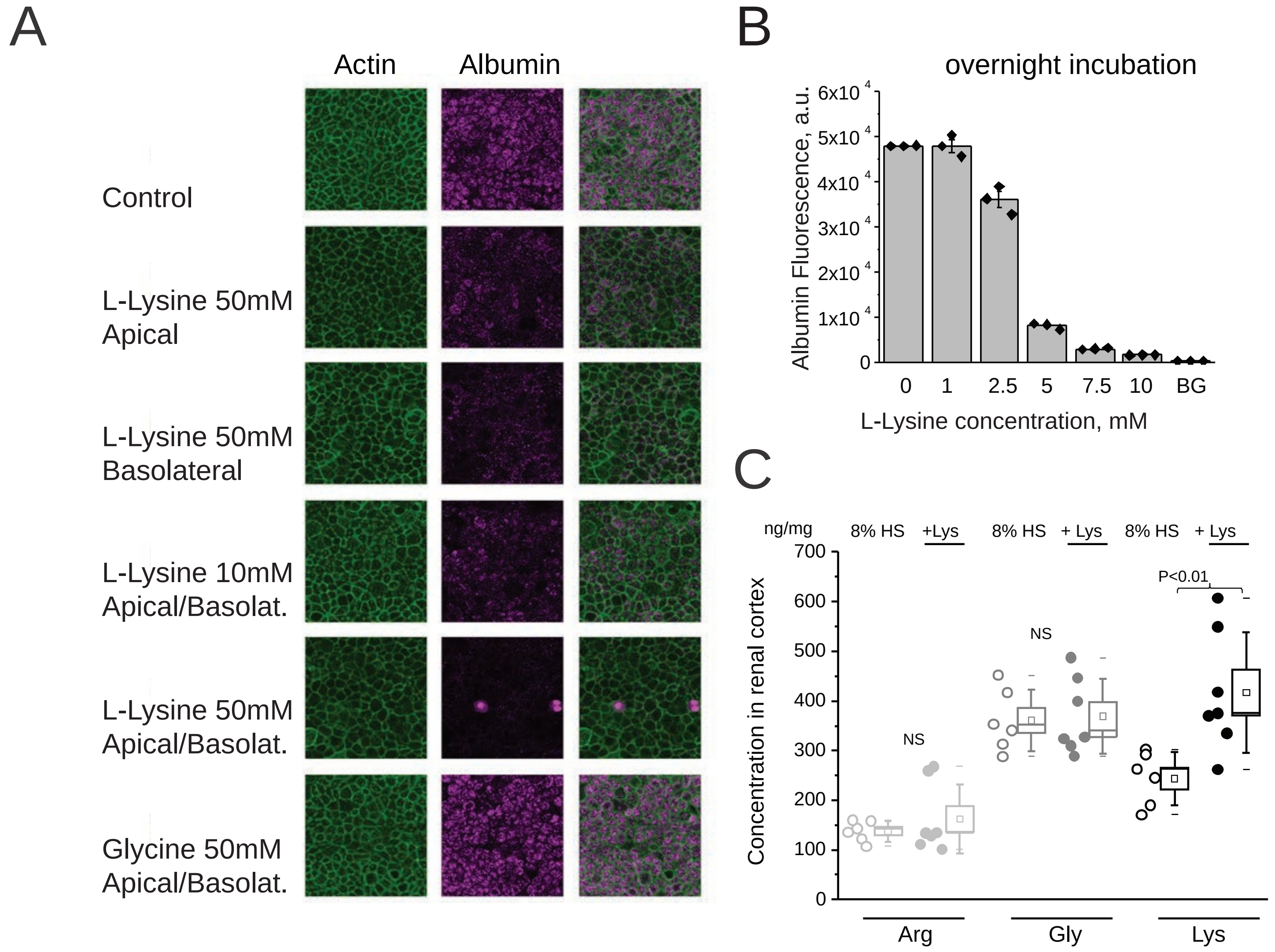

Supplemental Fig. 4: Albumin uptake in OK cells and amino acids in hypertensive kidneys. A. Immune fluorescence analysis of albumin uptake in OK cells with different treatment protocols from apical and basolateral. **B**. Concentration-dependent inhibition of albumin uptake with lower concentrations of Lysine administered overnight (~16h). N= 5 independent cell culture experiments, error bars = SEM **C**. Measurement of amino acids (Arginine, Glycine and Lysine) in hypertensive kidneys by HPLC. Two-tailed unpaired t-test.

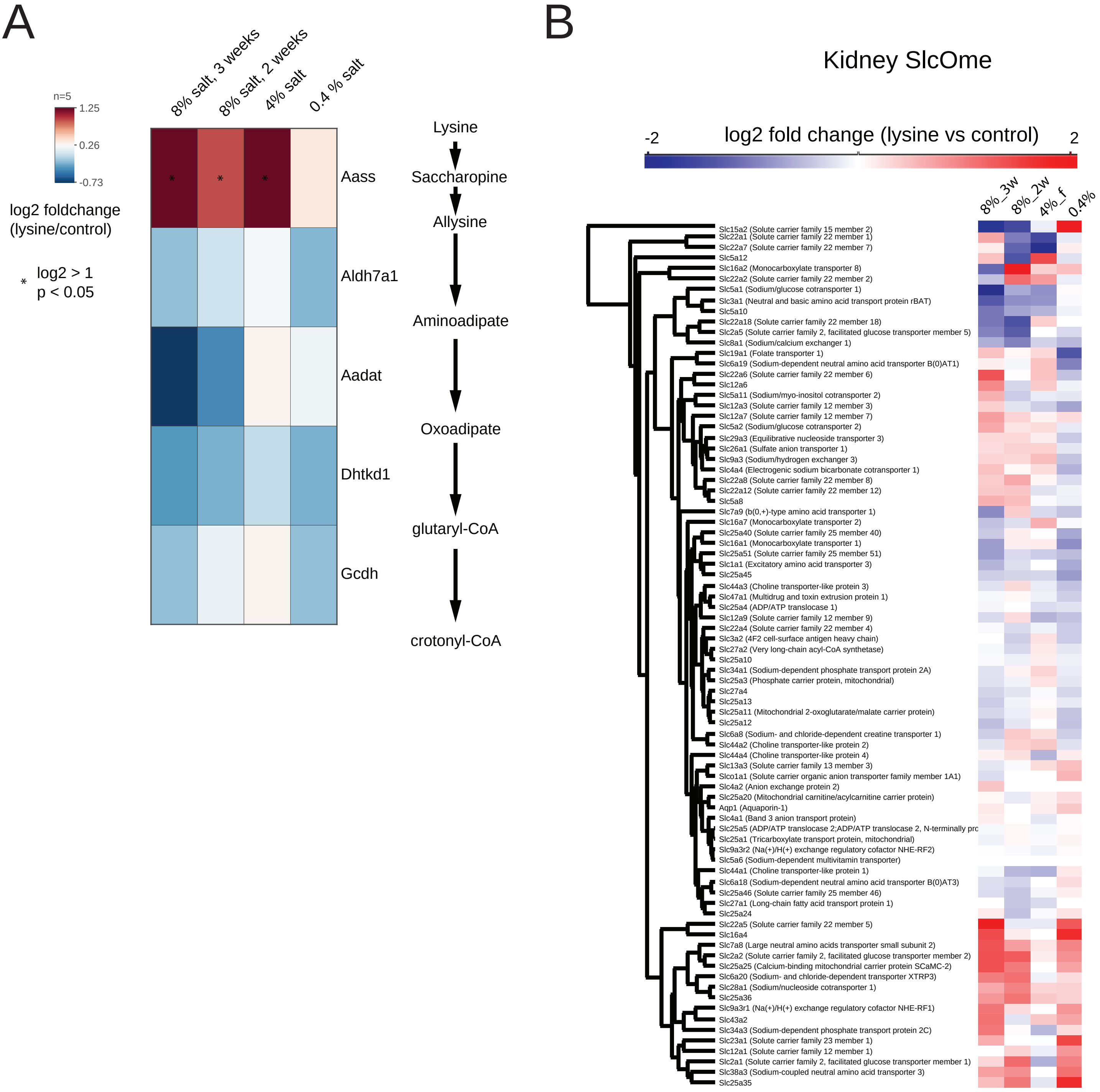

Supplemental Fig. 5: Proteomic analysis of lysine effects on the kidney. A. Proteomics analysis of kidney cortices treated with lysine. **B**. Regulation of Slc transporters with lysine in the hypertensive kidney.

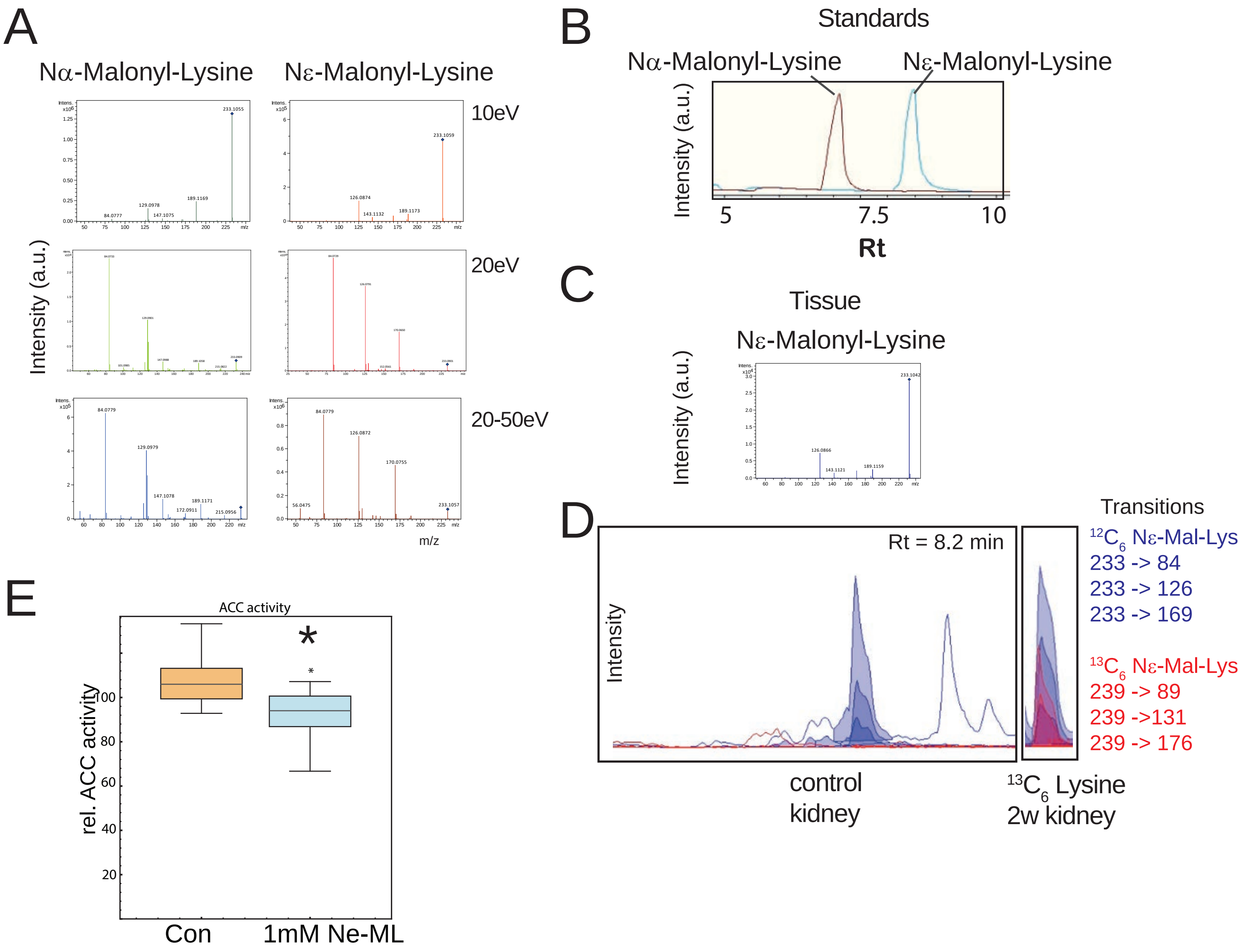

Supplemental Fig. 6: Chemical Characterization of Malonyl-lysine. **A.** MS2 tandem fragmentation spectra of malonyl-lysine compounds at different collision energies. **B.** Extracted MS1 basepeak chromatogram and retention time of compounds in HILIC chromatography. **C.** MS2 spectra from tissue. **D.** Targeted metabolomics assay from kidneys from unlabeled mice and mice fed with $^{13}C_6$ Lysine for 2 weeks demonstrates $^{13}C_6$ labeled malonyl-lysine fragments in isotope labeled kidney. **E**. Effect of 1 mM Nε-malonyl-lysine on ACC activity in lysates of OK cells.

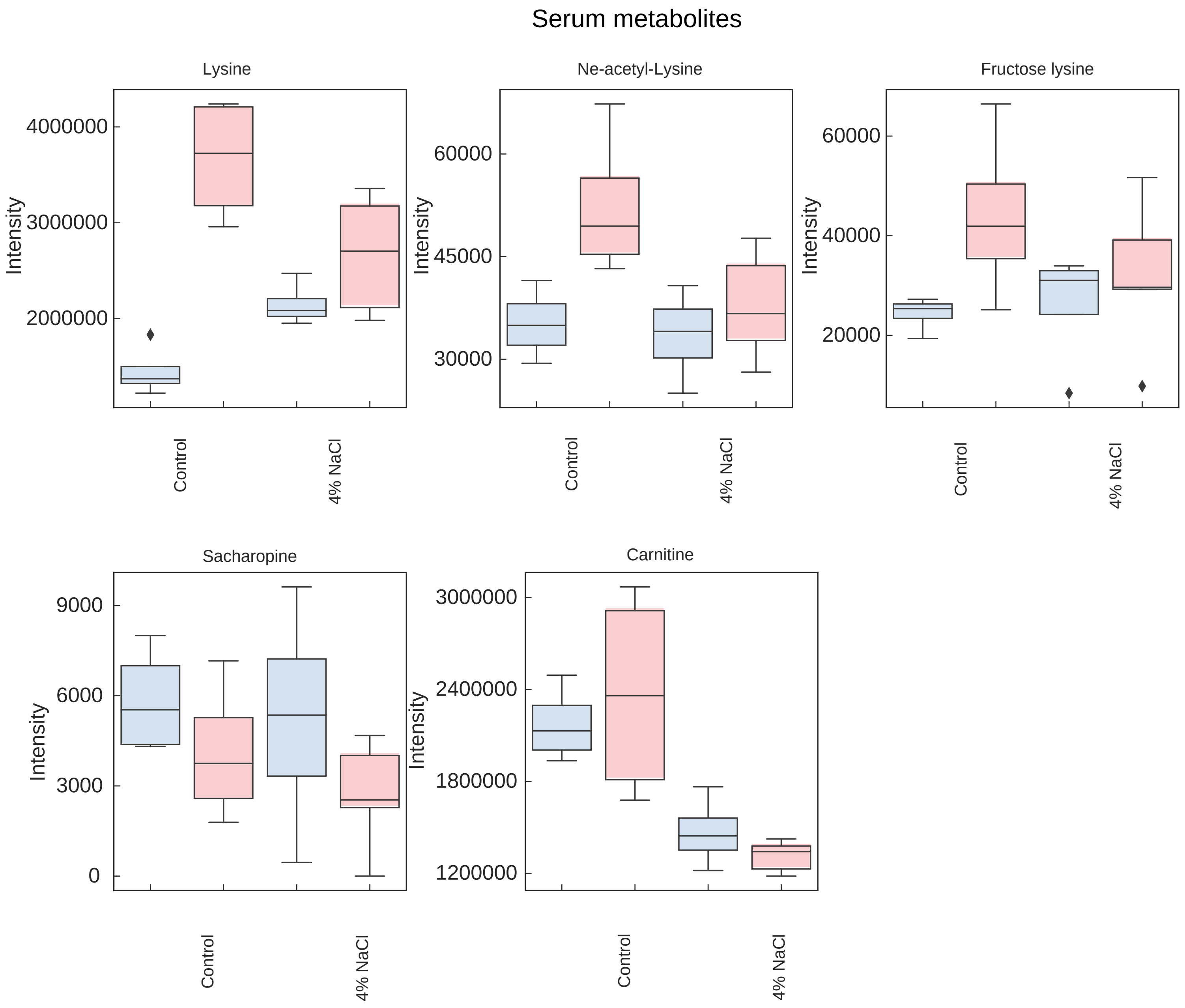

Supplemental Fig. 7: Metabolomic serum analysis of key lysine metabolites. Untargeted metabolomics analysis was performed for the respective lysine metabolites in serum from hypertensive and non-hypertensive rats and revealed no significant difference in lysine metabolites, except for significant increase of lysine in control with lysine treatment (n=5 per group, blue bars indicate control, red bars indicate lysine treated).

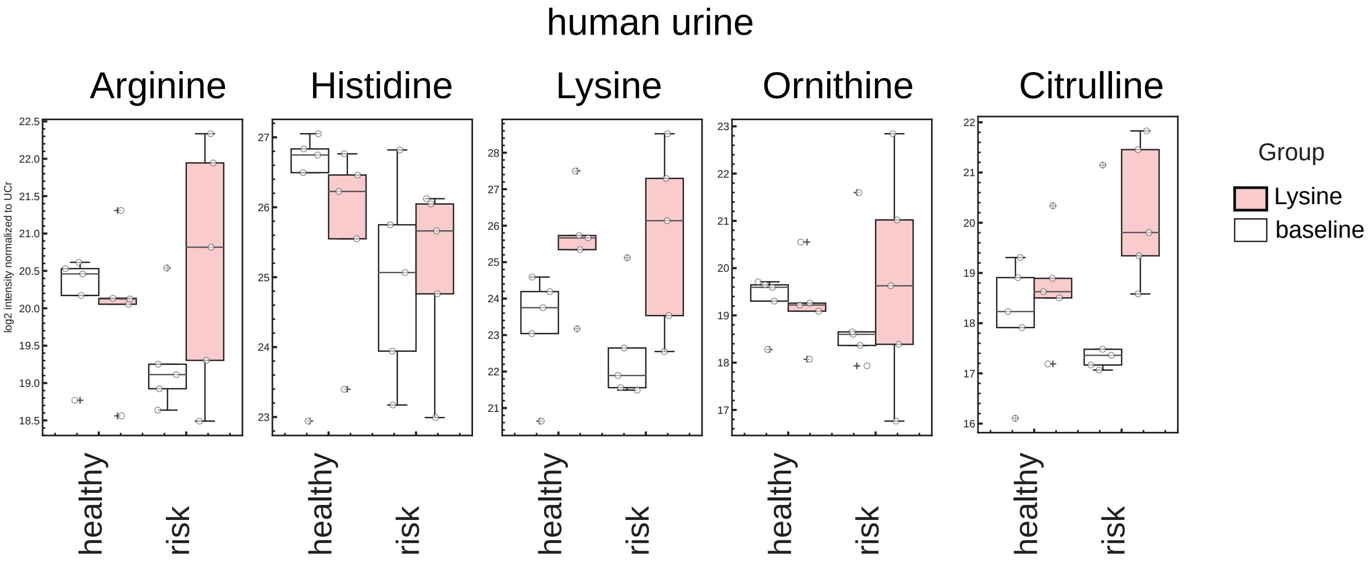

Supplemental Figure 8. Other positively charged (dibasic) amino acids in human urines with lysine treatment.
