# Supplementary material for: Accelerated lysine metabolism conveys kidney protection in salt-sensitive hypertension
Source: Nat Commun. 2022 Jul 14;13:4099. doi: 10.1038/s41467-022-31670-0 (PMC9283537; doi:10.1038/s41467-022-31670-0)
Supplement: Supplementary file 2 — Reporting Summary [file 41467_2022_31670_MOESM2_ESM.pdf]

nature portfolio

Corresponding author(s): Rinschen, Siuzdak, Staruschenko

Last updated by author(s): 4/12/2022

Reporting Summary

Nature Portfolio wishes to improve the reproducibility of the work that we publish. This form provides structure for consistency and transparency in reporting. For further information on Nature Portfolio policies, see our Editorial Policies and the Editorial Policy Checklist.

Statistics

For all statistical analyses, confirm that the following items are present in the figure legend, table legend, main text, or Methods section.

n/a	Confirmed	
[ ]	[x]	The exact sample size (*n*) for each experimental group/condition, given as a discrete number and unit of measurement
[ ]	[x]	A statement on whether measurements were taken from distinct samples or whether the same sample was measured repeatedly
[ ]	[x]	The statistical test(s) used AND whether they are one- or two-sided<br>*Only common tests should be described solely by name; describe more complex techniques in the Methods section.*
[x]	[ ]	A description of all covariates tested
[ ]	[x]	A description of any assumptions or corrections, such as tests of normality and adjustment for multiple comparisons
[x]	[ ]	A full description of the statistical parameters including central tendency (e.g. means) or other basic estimates (e.g. regression coefficient) AND variation (e.g. standard deviation) or associated estimates of uncertainty (e.g. confidence intervals)
[ ]	[x]	For null hypothesis testing, the test statistic (e.g. *F*, *t*, *r*) with confidence intervals, effect sizes, degrees of freedom and *P* value noted<br>*Give P values as exact values whenever suitable.*
[x]	[ ]	For Bayesian analysis, information on the choice of priors and Markov chain Monte Carlo settings
[x]	[ ]	For hierarchical and complex designs, identification of the appropriate level for tests and full reporting of outcomes
[x]	[ ]	Estimates of effect sizes (e.g. Cohen's *d*, Pearson's *r*), indicating how they were calculated

Our web collection on statistics for biologists contains articles on many of the points above.

Software and code

Policy information about availability of computer code

Data collection	The following softwares were used for data collection: OriginPro 2021b, XCMS online, MaxQuant v 1.5.3.3, Masshunter (Agilent, version 10.1) and Compass Hystar version 4.1 SR1.
Data analysis	Processing scripts are available through Github (see isotope selection approaches, for correlation based approach https://github.com/hpbenton/targeted_isotopes), and for mass-difference approach https://github.com/xdomingoal/isoTracker), doi: 10.5281/zenodo.6447658

For manuscripts utilizing custom algorithms or software that are central to the research but not yet described in published literature, software must be made available to editors and reviewers. We strongly encourage code deposition in a community repository (e.g. GitHub). See the Nature Portfolio guidelines for submitting code & software for further information.

Data

Policy information about availability of data

All manuscripts must include a data availability statement. This statement should provide the following information, where applicable:

- Accession codes, unique identifiers, or web links for publicly available datasets
- A description of any restrictions on data availability
- For clinical datasets or third party data, please ensure that the statement adheres to our policy

Proteomics data is available through the PRIDE/proteomExchange repository 73,74, http://www.ebi.ac.uk/pride . Project accession: PXD007749. Reviewer account details: Username: reviewer93072@ebi.ac.uk, Password: 9PpJ1Jz5. Project accession: PXD029232, Project DOI: 10.6019/PXD029232, Username: reviewer_pxd029232@ebi.ac.uk, Password: xSiq5yRB. Metabolomics data are available through Massive 75. MSV000089224, MSV000089223, ftp://MSV000089223@massive.ucsd.edu, password: Kidney, and ftp://MSV000089224@massive.ucsd.edu, password: Kidney. Uniprot RefSeq reference proteome databases from January 2017 (mouse) and from May 2020 (rat) were downloaded from www.uniprot.org.

Field-specific reporting

Please select the one below that is the best fit for your research. If you are not sure, read the appropriate sections before making your selection.

- [x] Life sciences
- [ ] Behavioural & social sciences
- [ ] Ecological, evolutionary & environmental sciences

For a reference copy of the document with all sections, see nature.com/documents/nr-reporting-summary-flat.pdf

Life sciences study design

All studies must disclose on these points even when the disclosure is negative.

Sample size	Our familiarity with the literature in this area and our expertise with the rodent species and the techniques used ensure that the fewest number of animals possible are used for study PMID: 33390052 PMID: 33046522 PMID: 24646854. We have performed a Power Analysis (SigmaPlot 12.0) to determine estimates of animals considering the variance of the measurements and the minimum changes we expect to observe. Male and Female difference was tested separately.
Data exclusions	No data were excluded. Only tissues without a complete isotope labeling set (Bladder, Heart, Hypophysis, BAT), were excluded from the isotope labeling study.
Replication	Replicated in more than 30 animals in at least 4-5 different batches of animals. Non-animal studies were repeated 3 times or more.
Randomization	Samples and animals were allocated to treatment in a random fashion. All mass spectrometry acquisitions were obtained in a random order. An exception was the analysis of isotope labeled tissue where samples were acquired in the order of labeling.
Blinding	Investigators were blinded to group allocation during data collection.

Reporting for specific materials, systems and methods

We require information from authors about some types of materials, experimental systems and methods used in many studies. Here, indicate whether each material, system or method listed is relevant to your study. If you are not sure if a list item applies to your research, read the appropriate section before selecting a response.

Materials & experimental systems

n/a	Involved in the study	
[ ]	[x]	Antibodies
[ ]	[x]	Eukaryotic cell lines
[x]	[ ]	Palaeontology and archaeology
[ ]	[x]	Animals and other organisms
[ ]	[x]	Human research participants
[x]	[ ]	Clinical data
[x]	[ ]	Dual use research of concern

Methods

n/a	Involved in the study	
[x]	[ ]	ChIP-seq
[x]	[ ]	Flow cytometry
[x]	[ ]	MRI-based neuroimaging

Antibodies

Antibodies used	1:1000 acetylated-lysine CST (9441), 1:1000 malonyl-lysine CST (#14942), 1:5000 beta-actin Genscript (A00702), 1:300 KIM1 Rat KIM-1Ab, 1:300, #AF3689, R&D Systems, 1:5000 HRP-conjugated secondary antibodies (mouse anti-rabbit Jackson Immunoresearch 211-032-171, rabbit anti-mouse Jackson Immunoresearch 211-035-109). The Megalin antibody was a homemade antibody by Franziska Theilig (no reference available).
Validation	We did not perform additional validation of the primary antibodies for the species and application, we can only note any validation statements on the manufacturer's websites. Acetylated lysine cst antibody and malonyl-lysine antibodies recognize modified lysine from various species.

Eukaryotic cell lines

Policy information about cell lines

Cell line source(s)	OK-P cells (RRID:CVCL_0472) are female Didelphis virginiana opossum proximal tubule cells. They were originally obtained from Moshe Levi (Georgetown University).
Authentication	RNASeq studies have confirmed that these cells are of opossum origin and most closely resemble the PT S1 segment (Eshbach et al PMID: 28615248; Park et al. PMID: 33192601)

Mycoplasma contamination	Cells were regularly tested for mycoplasma contamination using the Lonza mycoplasma detection kit and tests were negative.
Commonly misidentified lines (See ICLAC register)	None

Animals and other organisms

Policy information about studies involving animals; ARRIVE guidelines recommended for reporting animal research

Laboratory animals	SS/JrHsdMcwi, RRID:RGD_1579902, sex: male and female, age: 8 weeks<br>SHR rats: 8 week old male, RRID:RGD_61000, sex: male, age: 8 weeks<br>Male Bl6/N mice were 12 weeks old
Wild animals	No wild animals were used.
Field-collected samples	No field-collected samples were used.
Ethics oversight	All studies using D/SS rats were conducted at Medical College of Wisconsin and protocols were approved by the MCW Animal Care and Use Committees and were performed in accordance with the standards set forth by the NIH Guide for the Care and Use of Laboratory Animals (National Academies Press, 2011). All mice studies were conducted as previously described in accordance to the local regulations authorities (Regierungspraesidium Darmstadt, and local animal ethics committee at the MaxPlanck Institute Bad Nauheim).

Note that full information on the approval of the study protocol must also be provided in the manuscript.

Human research participants

Policy information about studies involving human research participants

Population characteristics	The healthy volunteers had no medical diagnosis and were 25-45 years old. The risk patients included 4 patients with unilateral kidney due to unilateral renal agenesis (2) and nephrectomy for kidney transplantation (2), and one patient with mild albuminuria (<1g/24h). None of the risk patients was obese, and age range was between 51-57. The patients were not controlled for other co-variate dependent characteristics (i.e. gender/sex).
Recruitment	Patients were recruited from the nephrology clinics. Since this is a proof-of principle BESH study, we did not evaluate other biases although they are likely to be present.
Ethics oversight	Human studies were done with accordance and approval of Internal Review Board at Medical College of Wisconsin and in the agreement of DHHS rules and regulations for human research. Informed consents was obtained from all study participants.

Note that full information on the approval of the study protocol must also be provided in the manuscript.
