# Supplementary material for: Accelerated lysine metabolism conveys kidney protection in salt-sensitive hypertension
Source: Nat Commun. 2022 Jul 14;13:4099. doi: 10.1038/s41467-022-31670-0 (PMC9283537; doi:10.1038/s41467-022-31670-0)
Supplement: Supplementary file 4 — Description of Additional Supplementary Files [file 41467_2022_31670_MOESM4_ESM.pdf]

Title: Supplemental Data 1:

Description: Molecular information of Lysine metabolites discovered by the mass-difference-based isotopologue selection approach.

Title: Supplemental Data 2:

Description: Molecular information of Lysine metabolites discovered by correlation-based extraction approach.

Title: Supplemental Data 3:

Description: Quantification of metabolites from kidney cortex from hypertensive and non-hypertensive D/SS rats with and without lysine treatment.

Title: Supplemental Data 4:

Description: Proteomic analysis of lysine effects on kidney cortex.
