# Supplementary material for: Accelerated lysine metabolism conveys kidney protection in salt-sensitive hypertension
Source: Nat Commun. 2022 Jul 14;13:4099. doi: 10.1038/s41467-022-31670-0 (PMC9283537; doi:10.1038/s41467-022-31670-0)
Supplement: Supplementary file 9 — Supplemental Data 5 [file 41467_2022_31670_MOESM9_ESM.docx]

| Cpd Name | Prec Ion | Prod Ion | Frag (V) | CE (V) | Cell Acc (V) | Ret Time (min) | Ret Window | Polarity |
| --- | --- | --- | --- | --- | --- | --- | --- | --- |
| 12C5-Alpha-ketogluterate | 145.01 | 101 | 166 | 4 | 4 | 2.4 | 1.2 | Negative |
| 12C6-Cis-aconitate | 173.01 | 85.1 | 166 | 12 | 4 | 3.4 | 1 | Negative |
| 12C6-Citrate_Isocitrate | 191.02 | 111 | 166 | 12 | 4 | 1.2 | 0.7 | Negative |
| 12C6-Citrate | 191.02 | 87 | 166 | 16 | 4 | 1.25 | 0.6 | Negative |
| 12C4-Fumarate | 115 | 71.1 | 166 | 4 | 4 | 1.95 | 1 | Negative |
| 12C6-Glucose | 179.05 | 89 | 166 | 4 | 4 | 0.75 | 0.4 | Negative |
| 12C6-Glucose | 179.05 | 59.1 | 166 | 16 | 4 | 0.75 | 0.4 | Negative |
| 12C6-Isocitrate | 191.02 | 117 | 166 | 12 | 4 | 1 | 0.5 | Negative |
| 12C3-Lactate | 89.02 | 43.2 | 166 | 8 | 4 | 0.95 | 0.6 | Negative |
| 12C4-Malate | 133.01 | 115 | 166 | 8 | 4 | 1.1 | 0.7 | Negative |
| 12C3-Pyruvate | 87.01 | 43.2 | 166 | 4 | 4 | 2 | 0.7 | Negative |
| 12C4-Succinate | 117.02 | 73.1 | 166 | 12 | 4 | 1.1 | 0.4 | Negative |
